# Tolerance of biofilm of a carbapenem-resistant *Klebsiella pneumoniae* involved in a duodenoscopy-associated outbreak to the disinfectant used in reprocessing

**DOI:** 10.1186/s13756-022-01112-z

**Published:** 2022-06-03

**Authors:** Melanie S. Brunke, Katharina Konrat, Christoph Schaudinn, Brar Piening, Yvonne Pfeifer, Laura Becker, Ingeborg Schwebke, Mardjan Arvand

**Affiliations:** 1 Unit for Hospital Hygiene, Infection Prevention and Control, Robert Koch Institute, Berlin, Germany; 2 Unit for Advanced Light and Electron Microscopy, Robert Koch Institute, Berlin, Germany; 3 Institute for Hygiene and Environmental Medicine, Charité - Universitätsmedizin Berlin, Berlin, Germany; 4 Unit for Nosocomial Pathogens and Antimicrobial Resistances, Robert Koch Institute, Wernigerode, Germany; 5 Hessisches Landesprüfungs- und Untersuchungsamt Im Gesundheitswesen, Dillenburg, Germany; 6 Department of Hygiene and Medical Microbiology, Institute of Hygiene, University of Heidelberg, Heidelberg, Germany

**Keywords:** Outbreak, Antimicrobial resistance, Disinfection, Peracetic acid, Duodenoscope, Reprocessing, Gram-negative, Carbapenemase, OXA-48

## Abstract

**Background:**

One possible transmission route for nosocomial pathogens is contaminated medical devices. Formation of biofilms can exacerbate the problem. We report on a carbapenemase-producing *Klebsiella pneumoniae* that had caused an outbreak linked to contaminated duodenoscopes. To determine whether increased tolerance to disinfectants may have contributed to the outbreak, we investigated the susceptibility of the outbreak strain to disinfectants commonly used for duodenoscope reprocessing. Disinfection efficacy was tested on planktonic bacteria and on biofilm.

**Methods:**

Disinfectant efficacy testing was performed for planktonic bacteria according to EN standards 13727 and 14561 and for biofilm using the Bead Assay for Biofilms. Disinfection was defined as ≥ 5log_10_ reduction in recoverable colony forming units (CFU).

**Results:**

The outbreak strain was an OXA-48 carbapenemase-producing *K. pneumoniae* of sequence type 101. We found a slightly increased tolerance of the outbreak strain in planktonic form to peracetic acid (PAA), but not to other disinfectants tested. Since PAA was the disinfectant used for duodenoscope reprocessing, we investigated the effect of PAA on biofilm of the outbreak strain. Remarkably, disinfection of biofilm of the outbreak strain could not be achieved by the standard PAA concentration used for duodenoscope reprocessing at the time of outbreak. An increased tolerance to PAA was not observed in a *K. pneumoniae* type strain tested in parallel.

**Conclusions:**

Biofilm of the *K. pneumoniae* outbreak strain was tolerant to standard disinfection during duodenoscope reprocessing. This study establishes for the first time a direct link between biofilm formation, increased tolerance to disinfectants, reprocessing failure of duodenoscopes and nosocomial transmission of carbapenem-resistant *K. pneumoniae*.

**Supplementary Information:**

The online version contains supplementary material available at 10.1186/s13756-022-01112-z.

## Background

One of the most prominent fast-spreading, multidrug-resistant nosocomial pathogens is *Klebsiella pneumoniae*. In Europe, carbapenemase-producing *K. pneumoniae* (CRKP) have been estimated to show the highest increase in the number of infections and attributable deaths between 2007 and 2015 compared to other antibiotic-resistant bacteria [1]. The epidemiology of CRKP shows a fast spread of highly successful, well-adapted strains in healthcare settings [2].

An important contributing factor to the successful nosocomial spread of CRKP is its ability to form biofilms [3]. In general, biofilms may resist cleaning and disinfection measures to a higher degree than planktonic bacteria and may thus serve as a reservoir for subsequent spread. *K. pneumoniae* biofilms have been found on hospital surfaces [4], sinks and drains [5] and, importantly, on medical devices such as endoscopes [6].

In the past decade, several outbreaks by CRKP were reported in which transmission was linked to contaminated duodenoscopes [7–9]. Duodenoscopes are flexible, complex instruments that are difficult to clean and made of sensitive materials that do not allow thermal disinfection [10–13]. Thorough cleaning and disinfection are critical to prevent transmission of microorganisms. Yet, in spite of strict adherence to cleaning and disinfection protocols, reprocessing failure of endoscopes has been frequently found to be involved in nosocomial transmissions and outbreaks of CRKP [13]. However, due to the lack of systematic monitoring and the limited sensitivity of diagnostic tools, the true extent of this problem remains unknown [14].

We investigated a CRKP strain which had been causative of an outbreak affecting thirteen patients in a hospital in Berlin in 2014. The endoscopy unit and a distinct type of duodenoscope were identified as the source. The outbreak investigation revealed that duodenoscope reprocessing had been performed by strictly following the manufacturer´s instructions. In this study we assessed the susceptibility of the outbreak strain to disinfectants commonly used for duodenoscope reprocessing to test whether increased tolerance to disinfectants may have contributed to the outbreak.

Currently, disinfection recommendations are based on experimental data obtained from reference type strains that are tested as planktonic cells in suspension or attached to a surface according to the international standards EN 13727 and EN 14561. The application of disinfectant efficacy testing to bacteria in biofilm has not been established yet.

We here investigated the efficacy of disinfection on planktonic bacteria and on biofilm, hypothesizing that the latter might display a higher, and thus clinically relevant tolerance to disinfectants. Efficacy testing was performed on: (1) planktonic cells in suspension, (2) planktonic cells fixed to a surface, and (3) cells embedded in biofilm.

For the latter part we used the Bead Assay for Biofilms which has been developed in our laboratory and has proven to be a reliable and robust method for testing the efficacy of disinfectants on biofilm-embedded bacteria [15]. We tested several disinfectants that are commonly used for disinfection of duodenoscopes in Germany, including glutaraldehyde (GA) and peracetic acid (PAA) [16, 17]. Our objective was to establish a direct link between biofilm formation, decreased susceptibility to disinfectants, and inadequate disinfection during reprocessing leading to a nosocomial outbreak.

### Description of the outbreak

Between May and November 2014, an outbreak of CRKP occurred in a hospital in Berlin affecting 13 patients. The outbreak strain was recovered from clinical specimens in eight patients (including blood cultures, wound swabs and tracheal aspirates), rectal screening cultures in five patients, two duodenoscopes and one gastroscope (16 isolates in total). Both duodenoscopes belonged to a brand that had previously been involved in other CRKP outbreaks [6, 7], and which was retracted by the manufacturer and redesigned thereafter. The reprocessing recommendations were also subsequently updated by the manufacturer. During the outbreak, duodenoscope reprocessing in the affected unit was performed according to the manufacturers’ instructions using a commercially available product containing PAA, with a working concentration of 0.15% (w/w) PAA and 10 min exposure time at 25 °C in an automated washer-disinfector. In response to this outbreak, parts of the duodenoscopes were replaced with newly designed elements by the manufacturer and the duodenoscope reprocessing procedure was changed to a GA-based disinfection (10 min exposure time, 55 °C). No further cases occurred after these changes.

### Molecular characterization of the outbreak strain

Pulsed-field gel electrophoresis (PFGE)-based typing confirmed that the CRKP isolates obtained during the outbreak belonged to the same strain (Additional file 1: Fig. S1A). As a representative of the outbreak strain, the *K. pneumoniae* isolate 886/14 obtained from a clinical sample of a patient was further analysed. Antimicrobial susceptibility testing revealed that the isolate was resistant to cephalosporins and carbapenems and susceptible only to amikacin, colistin and tigecycline (Additional file 2: Table S1). Carbapenemase production was proven by modified Hodge test and was encoded by the *bla*_OXA48_ gene located on a self-conjugable IncL/M megaplasmid, determined by PCR-based Sequencing as described previously [18] (Additional file 1: Fig. S1B). The outbreak strain was subjected to whole genome sequencing. Multilocus Sequence Typing (MLST) using the MLST tool revealed that the outbreak strain belonged to the sequence type (ST)101 [19]. The complete antimicrobial susceptibility profile and detailed molecular characterization are provided in Additional file 2: Material 1 and Table S2.

### Efficacy testing of disinfectants on the outbreak strain

We investigated the susceptibility of the outbreak strain by initially testing four disinfectants that are commonly used for the reprocessing of duodenoscopes: hydrogen peroxide (H_2_O_2_), GA, isopropanol and PAA. For comparison, a *K. pneumoniae* type strain (ATCC 13883) was tested in parallel. Disinfection was defined as ≥ 5log_10_ reduction in recoverable mean CFUs. Experimental conditions, disinfectants and neutralizers are listed in Additional file 2: Table S3.

#### Testing of planktonic cells in suspension

As a first step, efficacy testing on planktonic cells in suspension was performed using the suspension test according to EN 13727 [20]. For H_2_O_2_, GA and isopropanol we found similar results for disinfection of the outbreak strain and the type strain (Table 1). While the latter three substances completely inactivated both strains at similar concentrations, we found a slight difference between the outbreak strain and the type strain with regard to the efficacy of PAA. The PAA concentration needed for disinfection of the outbreak strain was six-fold higher than the concentration needed for the type strain (0.003% versus ≤ 0.0005% (*w/v*) PAA) (Table 1). Still, both strains were sensitive to PAA in the suspension test. Since PAA was the disinfectant used in the respective hospital for endoscope reprocessing at the time of the outbreak the effect of PAA was further investigated using two additional tests, as described below.

**Table 1:** Results of efficacy testing by suspension test of four disinfectants

Disinfectant	Exposure time (min)	*K. pneumoniae* ATCC 13883	*K. pneumoniae* outbreak strain
H_2_O_2_	5	5 % (*w/v*)	5 % (*w/v*)
Glutaraldehyde	10	0.04 % (*w/v*)	0.04 % (*w/v*)
Isopropanol	1	30 % (*w/v*)	30 % (*w/v*)
Peracetic acid	10	≤ 0.0005 % (*w/v*)	0.003 % (*w/v*)

Conditions resulted in ≥ 5log10 reduction in CFU of *K. pneumoniae* strains are presented

#### Testing of surface-fixed planktonic cells

**Fig. 1:**
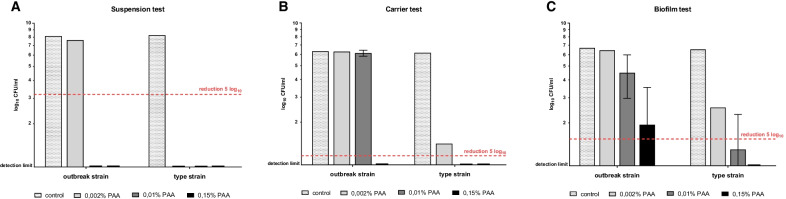
Efficacy of peracetic acid (PAA) using three different disinfectant testing methods. Bars show mean recovered CFUs after 10 min exposure to different concentrations of PAA of the *K. pneumoniae* outbreak strain and the type strain that were tested as: (**a**) planktonic cells (suspension test, EN 13727) (**b**) surface-fixed planktonic cells (carrier test, EN 14561) and (**c**) biofilm (Bead Assay for Biofilms). Disinfection was defined as ≥ 5log_10_ reduction in mean recoverable CFUs and is marked by a dashed red line in the respective assays. At comparable PAA concentrations, the outbreak strain shows a higher number of recovered CFUs than the type strain in all three models. Experiments were performed in triplicates

Efficacy testing for PAA on surface-fixed bacteria was performed using the quantitative carrier test for evaluation of bactericidal activity for medical instruments (instrument disinfection) according to EN 14561 [21]. At 10 min exposure time, higher concentrations were required for the inactivation of both strains as compared to the suspension test (Fig. 1). For surface-fixed bacteria, PAA concentrations of 0.01% and 0.15% were required for disinfection of the type strain and the outbreak strain, respectively. Still, complete inactivation was achieved for both strains in this assay at the PAA concentration used in duodenoscope reprocessing, i.e. 0.15%.

#### Testing bacteria in biofilm

**Fig. 2:**
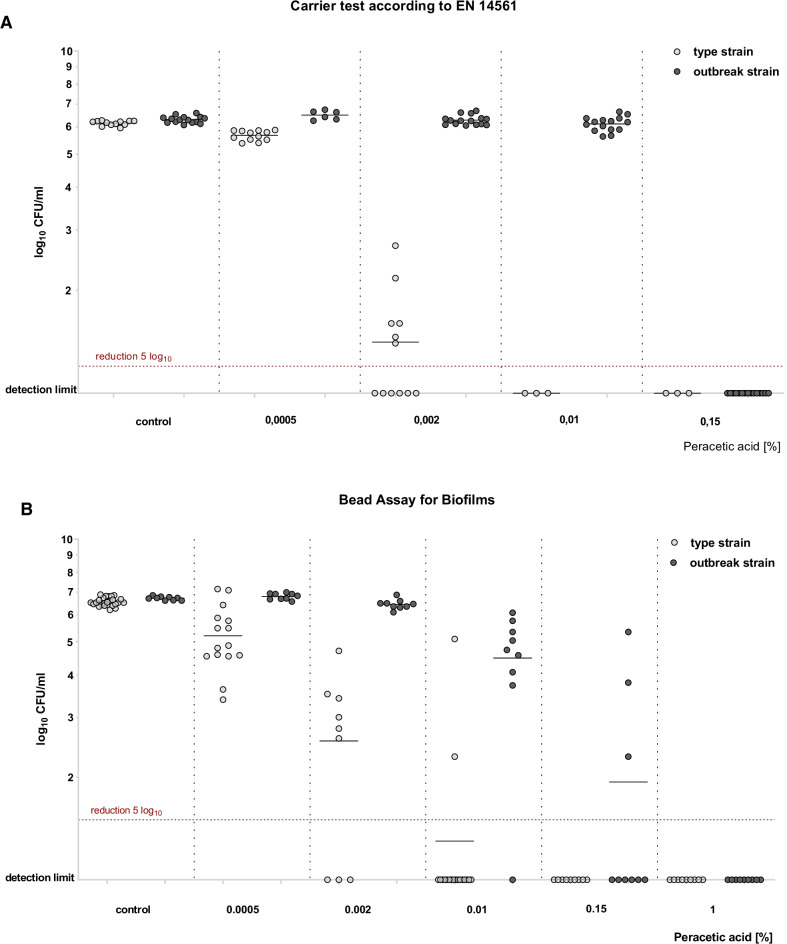
Effect of PAA on *K. pneumoniae* strains as surface-fixed cells (**a**) and biofilm (**b**). Scatter Plot with each dot representing recovered CFUs for a replicate after 10 min exposure to PAA. Disinfection was defined as ≥ 5log_10_ reduction in mean recoverable CFUs (dashed red line). The horizontal lines show the mean value for the respective replicates. In the biofilm model, 0.15% PAA, the concentration used in duodenoscope reprocessing, was not sufficient to achieve disinfection of the outbreak strain

We next assessed the efficacy of PAA on 24-h old biofilm of the outbreak strain and the type strain using the Bead Assay for Biofilms [15]. An even higher concentration of PAA was required to achieve disinfection of both strains (Fig. 1). Only at a PAA concentration of 1%, which is markedly higher than the concentration used for duodenoscope reprocessing (0.15%), did we observe a ≥ 5log_10_ CFU reduction of the outbreak strain in all biological replicates (Fig. 2). In contrast, the disinfection of the type strain was achieved at a lower concentration, i.e. 0.15% PAA.

## Discussion

We investigated a CRKP strain responsible for an outbreak in a duodenoscopy unit and found a clinically relevant increased tolerance towards PAA-based disinfection. Since PAA was used for duodenoscopes reprocessing at the time of the outbreak, this factor may be a key feature contributing to the nosocomial transmission of the outbreak strain to several patients, even though the reprocessing unit strictly followed standard disinfection protocols. Of note, the outbreak strain displayed tolerance to PAA, which was used for reprocessing, but not to the other disinfectants included in the initial testing, suggesting that the enhanced tolerance to PAA confers a specific advantage to the outbreak strain.

Bacteria in biofilm have previously been shown to be less susceptible to disinfectants than surface-fixed planktonic bacteria [15, 22]. Our data confirm these observations and show the clinical impact of this feature, meaning that biofilm of the outbreak strain could not be inactivated by the PAA concentration deemed as safe for routine reprocessing. It should be noted that biofilm of the type strain was successfully inactivated by the PAA concentration used in duodenoscope reprocessing, suggesting that this trait may be a unique feature of the outbreak strain.

The CRKP outbreak strain characterized in this study belongs to the epidemic ST101, a highly successful clonal lineage that often comprises carbapenemase-producing isolates [2, 23, 24]. Future research is needed to evaluate additional isolates of the ST101 lineage and maybe other epidemic CRKP lineages for tolerance to disinfectants.

Reprocessing of complex medical instruments such as duodenoscopes is a critical issue in infection prevention and control (IPC). It has been hypothesized that transmissions of pathogens by contaminated endoscopes may be severely underreported [14]. As a disinfectant, PAA is valued for its efficacy and is widely accepted by personnel due to safe handling [25]. Still, in spite of strict adherence to best practice, several CRKP outbreaks have been linked to thermolabile endoscopes [7–9]. Interestingly, an outbreak similar to the one described here involving a *K. pneumoniae* ST101 (OXA-48, CTX-M-15) strain associated with duodenoscopes occurred in another hospital in Berlin in 2012 [6], indicating that problems with CRKP and contaminated endoscopes are more common than generally assumed. *K. pneumoniae* and other biofilm producing species such as *Pseudomonas aeruginosa* and *Acinetobacter baumannii* were recently shown to survive the PAA-based duodenoscope reprocessing, with increasing colony counts after each reprocessing cycle over the course of one working day [26]. Our study shows that even correct reprocessing of endoscopes may result in a residual risk for nosocomial transmission of pathogens. Higher PAA concentrations that could efficiently inactivate biofilms such as those formed by the outbreak strain described here would inflict damage on the sensitive materials of duodenoscopes. The formation of miniscule cracks, for example in the duodenoscopes mantle, would provide additional niches for biofilm formation and persistence.

Our study has some limitations. First, we assessed only one outbreak isolate and compared it with a type strain. Future investigation of additional clinical CRKP isolates, including highly successful clonal lineages, is needed to assess whether the decreased susceptibility to disinfectants observed in the outbreak strains biofilm is a unique feature or might represent a general adaptive step of nosocomial strains to withstand continued exposure to disinfectants. Second, since the outbreak ended as both the duodenoscope model used and the disinfection procedure applied were changed, we cannot say which step was the most effective one in terminating the outbreak.

## Conclusion

In conclusion, we found that biofilm of the *K. pneumoniae* outbreak strain was tolerant to standard disinfection conditions that are currently established for duodenoscopes reprocessing. We consider the markedly increased tolerance of the outbreak strains' biofilm to PAA to be a major factor contributing to the outbreak. This study establishes for the first time a direct link between biofilm formation, increased tolerance to disinfectants, reprocessing failure of duodenoscopes and nosocomial transmission of *K. pneumoniae*. Bacterial biofilms must be considered systematically to avoid underexposure of bacterial pathogens to disinfectants, resulting in selective pressure towards development of tolerant strains.

## Supplementary Information

**Additional file 1**. Fig. S1A.

**Additional file 2**. Supplemental Material.

## Data Availability

The outbreak strain may be provided upon request to M.A. through a material transfer agreement. The authors will share data on request.
