# Supplementary figures and images for: Tolerance of biofilm of a carbapenem-resistant Klebsiella pneumoniae involved in a duodenoscopy-associated outbreak to the disinfectant used in reprocessing
Source: Antimicrob Resist Infect Control. 2022 Jun 3;11:81. doi: 10.1186/s13756-022-01112-z (PMC9164365; doi:10.1186/s13756-022-01112-z)

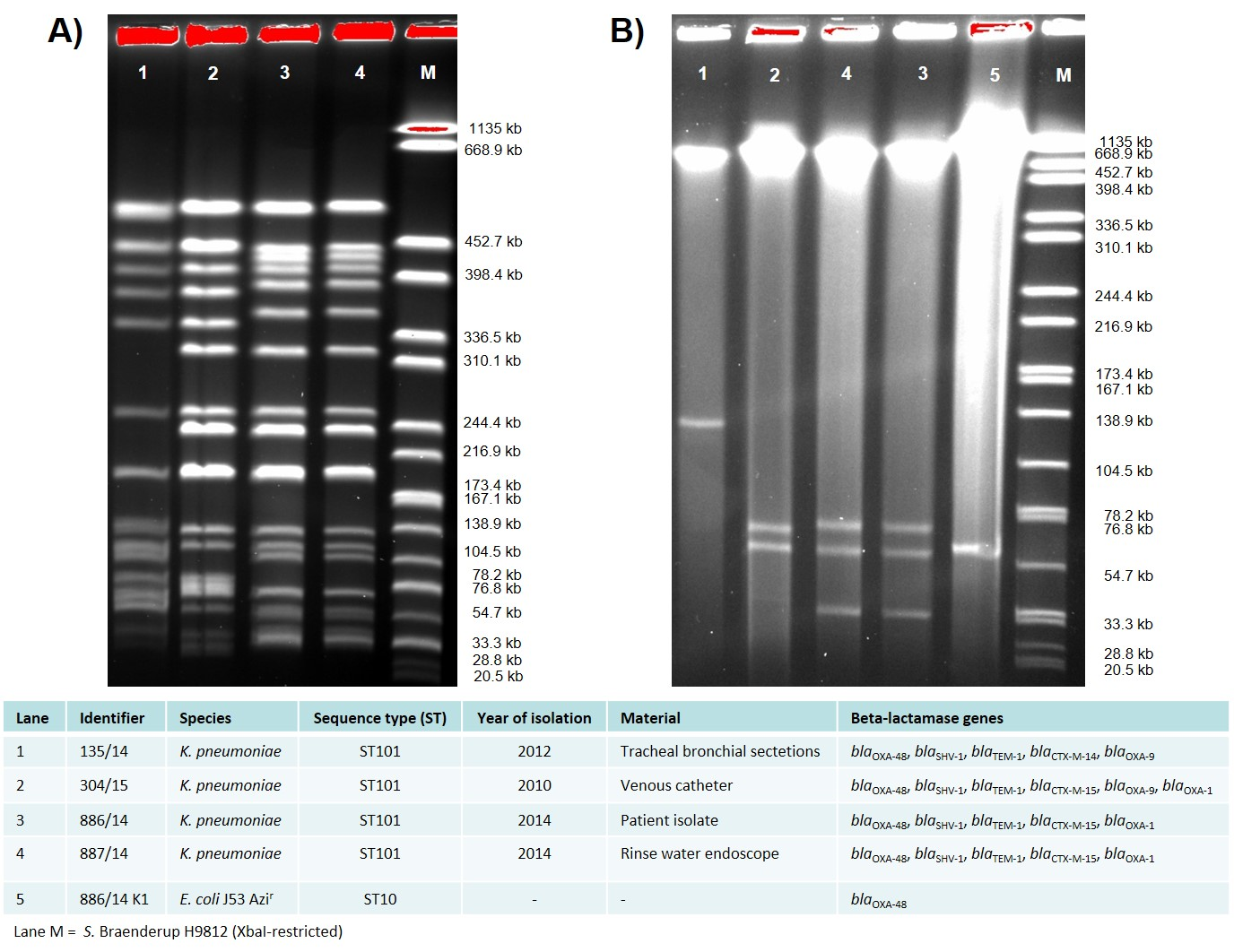

Supplement: Supplementary file 1 — Additional file 1. Fig. S1A. [file 13756_2022_1112_MOESM1_ESM.png]
