# Supplementary material for: Tolerance of biofilm of a carbapenem-resistant Klebsiella pneumoniae involved in a duodenoscopy-associated outbreak to the disinfectant used in reprocessing
Source: Antimicrob Resist Infect Control. 2022 Jun 3;11:81. doi: 10.1186/s13756-022-01112-z (PMC9164365; doi:10.1186/s13756-022-01112-z)
Supplement: Supplementary file 2 — Additional file 2. Supplemental Material. [file 13756_2022_1112_MOESM2_ESM.docx]

**Supplemental Material**

**Supplementary Material 1: Methods**

**Microbiological and molecular characterization**

Two *K. pneumoniae* isolates, one from a patient (886/14 chosen as a representative of the outbreak strain) and one from rinse water of the endoscope (isolate 887/14) were characterized. Genetic relationship of *K. pneumoniae* clinical isolates was confirmed by XbaI-restriction and pulsed-field gel electrophoresis (PFGE) [26]. Antibiotic susceptibilities were detected using broth microdilution, automated testing (supplementary table 1) and gradient tests (Etest, bioMérieux) with interpretation according to EUCAST v12.0 (https://www.eucast.org). Carbapenemase production was assessed by modified Hodge test [28]. Transfer of carbapenem resistance was tested for isolate 886/14 by broth mating and subsequent PFGE [29]. PCR-based screening for resistance genes and sequencing was performed as described previously including beta-lactamase genes and plasmid mediated fluoroquinolone resistance genes (supplementary table 2) [30]. Isolate *K. pneumoniae* 886/14 was subjected to whole genome sequencing. Sequencing libraries were prepared using the Nextera XT Kit (Illumina) and sequenced on an Illumina Miseq using v3 chemistry (2 × 300 bp) according to the manufacturer’s protocol. The data were analyzed using ResFinder [31], PlasmidFinder and the MLST tool [19]. Determination of capsular type of the outbreak strain was conducted by *wzi* gene sequencing [32] and using the websites of the Pasteur Institute (http://bigsdb.pasteur.fr/). The genome was screened for the presence of the following virulence genes or gene clusters using the reference databank of the Pasteur Institute: *mrk*, *ybt, clb, iro, iuc, kfu*, *rmpA* and *rmpA2* (Geneious v10, Biomatters).

**Disinfection efficacy testing**

*K. pneumoniae* was cultivated in Tryptic Soy Broth at 37°C. Disinfection experiments were performed at 20°C. The neutralizing agents (Supplementary Table 3) were evaluated as described previously [15]. The **quantitative suspension test** was performed according to EN 13727 without organic load [20]. Disinfectant dilutions (0.9mL) were mixed with 0.1mL bacterial suspension. Controls were treated with sterile H_2_O. After exposure, samples were diluted 1:10 in neutralizer.

The **quantitative carrier test** was performed according to EN 14561 [21]. Disinfection efficacy under practical conditions with organic load (bovine serum albumin and sheep erythrocytes) was evaluated using frosted glass carriers. 50µL of bacterial test suspension were dried (60 min at 30°C) and carriers were immersed in 10mL of PAA at the respective concentration (Supplementary table 3) for 10 min. Controls were treated with hard water instead of disinfectant. Subsequently, carriers were transferred to 10mL of neutralizer, and bacteria were detached mechanically and quantified.

Biofilm of *K. pneumoniae* strains was cultivated for 24h on glass beads as previously described [15]. For disinfection, biofilm was treated with PAA for 10 min [15]. Controls were treated with hard water. All experiments were performed with three technical replicates.

**Supplementary table S1. Antibiotic susceptibilities of the outbreak strain *K. pneumoniae* 886/14.**

| **Isolate (material)** |  | **Patient**  **„Outbreak strain“** | **Endoscope rinsing water** |
| --- | --- | --- | --- |
| **Internal identifier** |  | **886/14** | **887/14** |
| **Broth microdilution (EUCAST v12.0)** | | | |
| AMP | Ampicillin | >16 | > 16 |
| CTX | Cefotaxime | >16 | >16 |
| CAZ | Cetftazidime | > 32 | > 32 |
| FOX^1^ | Cefoxitin | > 32 | > 32 |
| GEN | Gentamicin | > 8 | > 8 |
| AMK | Amikacin | 4 | 4 |
| STR^1^ | Streptomycin | ≤4 | ≤4 |
| NAL^1^ | Nalidixinsäure | > 32 | > 32 |
| CMP | Chloramphenicol | 16 | 16 |
| TET^1^ | Tetracycline | 8 | 8 |
| CIP | Ciprofloxacin | > 64 | > 64 |
| COL | Colistin | ≤0,5 | ≤0,5 |
| MPM | Meropenem | > 8 | 8 |
| SXT | Sulfmethoxazole/Trimethoprim | 128 | 128 |
| **Etest** | | | |
| IPM | Imipenem | 8* | 8* |
| MPM | Meropenem | (>)32 | (>32) |
| ETP | Ertapenem | >32 | >32 |
| **VITEK 2 card AST N248** | | | |
| PIP | Piperacillin | > 64 | > 64 |
| ATM | Aztreonam | > 32 | > 32 |
| PTZ | Piperacillin/Tazobactam | > 64 | > 64 |
| IPM | Imipenem | > 8 | > 8 |
| CTX | Cefotaxime | >32 | >32 |
| CAZ | Ceftazidime | >32 | >32 |
| CEF | Cefepime | >32 | >32 |
| GEN | Gentamicin | > 8 | > 8 |
| TOB | Tobramycin | > 8 | > 8 |
| AMK | Amikacin | ≤2 | ≤2 |
| FOS | Fosfomycin | 32 | 32 |
| TIG | Tigecycline | 1 | 1 |
| COL | Colistin | ≤0,5 | ≤0,5 |
| MOX | Moxifloxacin | > 4 | > 4 |
| CIP | Ciprofloxacin | > 2 | > 2 |
| MPM | Meropenem | > 8 | > 8 |
| SXT | Sulfmethoxazole/Trimethoprim | 64 | 64 |

grey=resistant; ^1^ no clinical breakpoints but ECOFFs available for *Enterobacterales* (EUCAST; https://www.eucast.org/mic_distributions_and_ecoffs/); * colony growth in inhibition zone (presence of resistant subpopulations); minimum inhibitory concentrations (MICs) are given im mg/L.

**Supplementary table S2.** **Detected resistance genes of the outbreak strain *K. pneumoniae* 886/14**

| **Resistance genes mediating resistance to:** | **Strain *K. pneumoniae* 886/14 “outbreak strain”** |
| --- | --- |
| Beta-lactams | *bla*_OXA-48_ |
|  | *bla*_OXA-1_ |
|  | *bla*_SHV-1_ |
|  | *bla*_TEM-1A_ |
|  | *bla*_CTX-M-15_ |
| Aminoglycosides (fluoroquinolones) | *aac(3)-IIa* |
|  | *aac(6’)-Ib-cr* |
| Quinolones | *oqxA, oqxB* |
| Phenicols | *catB3* |
| Trimethoprim | *dfrA14* |

Genes were detected by analysis of whole genome sequence data using ResFinder [31].

**Supplementary table S3. Experimental conditions, disinfectants and neutralizers.**

| **Disinfectant** | **Method** | | **Tested concentrations** *(w/v)* | **Exposure time** | | | **Neutralizer**  (0.1M, pH7) |  |
| --- | --- | --- | --- | --- | --- | --- | --- | --- |
| **H_2_O_2_ 30%** | Suspension test | | 1% - 5% | 5 min | | | Catalase in phosphate buffer |  |
| **Glutaraldehyde 25%** | Suspension test | | 0.01% - 0.07% | 10 min | | | 0.5% Sodium sulfite in phosphate buffer |  |
| **Isopropanol 99.8%** | Suspension test | | 20% and 30% | 1 min | | | Phosphate buffer |  |
| **Peracetic acid 40%** (Wofasteril, Kesla Hygiene, Bitterfeld-Wolfen, Germany) | Suspension test  Carrier test  Bead Assay for Biofilms | 0.0004% – 0.006%  0.0005% – 0.15%  0.0001% – 1% | | | 5 and 10 min  10 min  10 min | 0.5% Sodium sulfite in phosphate buffer | | |
